# The paradox of convenience: how information overload in mHealth apps leads to medical service overuse

**DOI:** 10.3389/fpubh.2024.1408998

**Published:** 2024-11-28

**Authors:** Lingling Zhong, Junwei Cao, Fengtao Xue

**Affiliations:** School of Business, Yangzhou University, Yangzhou, China

**Keywords:** mHealth apps, information overload, overuse of medical services, health belief model, PLS-SEM

## Abstract

**Background:**

Mobile health applications (mHealth) have become an indispensable tool in the healthcare industry to provide users with efficient and convenient health services. However, information overload has led to significant information overload problems in mHealth applications, which may further lead to overuse of medical services.

**Methods:**

The purpose of this study was to explore the relationship between information overload and overuse of medical services in mHealth applications through health belief model (HBM). Data were collected from 1,494 respondents who were sampled through a simple random approach. A structured questionnaire was used as the instrument for data collection from mobile APP users in Guangdong Province between February 4, 2024, and February 20, 2024. Structural equation modeling (SEM) was used to analyze the data to investigate the effects of information overload on users’ perceived severity, susceptibility, treatment benefits, barriers, self-efficacy, and action cues, which further influence the overuse of health care services.

**Results:**

The study found that information overload significantly affected users’ perceived severity, susceptibility, treatment benefits, barriers, self-efficacy, and action cues, and subsequently affected overuse of health care services. These results provide valuable insights for mHealth application developers, healthcare providers, and policy makers.

**Conclusion:**

This study highlights the importance of effectively managing information delivery in mHealth applications to reduce the risk of overuse of healthcare services. The study not only highlights the dark side of information overload in mHealth applications, but also provides a framework to understand and address the challenges associated with information overload and service overuse in the mHealth context.

## 1 Introduction

In the medical field, adhering to the principle of “providing 100% of what is needed and avoiding 100% of what is unnecessary” is of paramount importance (1). This study believes that the definition of overuse of medical services can be adopted by S Brownlee et al., that is, overuse of medical services can be considered to occur continuously. At one end of the continuum, there are tests and treatments that, if applied to the right patients, are generally beneficial; At the other end of the continuum, services are completely ineffective or pose such a high risk of harm to all patients that they should never be provided (2). Recently, as the medical community increasingly embraces the “less is more” philosophy, the overuse of medical services has emerged as a prominent issue (3). Overutilization not only leads to financial burdens and excessive consumption of human resources but also exacerbates the strain on healthcare systems, potentially resulting in overemphasis on healthy individuals and neglect of patients. This phenomenon intensifies health inequalities, adversely affecting both individuals who require treatment and those who do not (4). For instance, in the United States, approximately 20.6% of medical services are considered unnecessary, including 22.0% of prescription medications Brownlee and 24.9% of tests (5). Overutilization has become one of the primary drivers of rising healthcare costs in the U.S. (6), with billions of dollars wasted annually, much of which does not improve health outcomes (7). Similarly, in China, the overuse of medical services is a key factor in the significant growth of healthcare expenses (8).

In the current healthcare system, the issue of limited resources and overuse is one of the major challenges facing health policymakers. Overuse of medical services not only increases the cost of care, but also reduces the quality of care, and in many cases, patients can make better decisions if they have enough information (9). Therefore, identifying and addressing the precursors to medical service overuse in the current technological context has become a critical issue.

Currently, numerous scholars have explored the factors influencing patients’ overutilization of medical services from various perspectives. First, the characteristics of hospitals are believed to be closely related to patients’ overuse of medical services. For instance, a study in the United States using cluster analysis found that overutilization of medical services among insured populations is more severe in non-teaching and for-profit hospitals, especially in the Southern United States (10). Additionally, personal characteristics of physicians, such as age, training status, and research activities, have been proven to have a direct impact on medical overuse (11). Regional policies and cultural orientations are also associated with medical overuse; some studies indicate that financial incentives and medical culture in a region are drivers of excessive use of medical services, while areas with higher voluntary rates of primary care services tend to have lower levels of service overutilization (12).

Secondly, low-value medical services are considered a key driving factor in the overutilization of medical services. In the healthcare sector, when users perceive the risks to outweigh the benefits, there may be a demand for low-value medical services (13). These services often focus on low-value medical tests, and a mismatch between patients’ demand for aggressive testing and testing capabilities leads to the overutilization of medical services (14). Some studies suggest that preventing low-value testing from the initial stages of medical care through effective collaboration between physicians and patients can reduce the overuse of healthcare services (15).

Moreover, patients’ psychological mechanisms and information retrieval methods are closely related to the overutilization of medical services. Literature reviews indicate that American patients’ reliance on anecdotal evidence, the use of diagnostic labels, and the pursuit of maximizing medical efficacy all influence their behavior of overutilizing medical services (6). Other study points out that the contradiction between patients’ cognitive uncertainty and the demand for maximizing medical efficacy is driving the occurrence of overutilization of medical services (14).

These studies emphasize that medical information and services provided by healthcare providers, such as hospitals and doctors, significantly influence users’ health behaviors but can also contribute to patients’ overutilization of medical services. In the post-pandemic era, Mobile health apps (mHealth apps) have become an indispensable tool in the medical industry, providing users with efficient and convenient health services. Mobile health applications can be defined as standalone software that exists on smart devices (such as smartphones, tablets, computers), and unlike other applications, mobile health applications are able to function as clinical tools in medical practice and are widely used for medical education, point-of-care services, direct interaction with patients, and as clinical reference resources (16). Thus, as patients increasingly turn from in-person doctor visits to online channels for medical services, mHealth becomes the primary choice. However, this shift also means that mHealth could become a new factor contributing to the overutilization of medical services. Studies indicate that specific mHealth applications, particularly those related to mental health, could lead to overdiagnosis because these apps often inappropriately use general screening tools as diagnostic instruments, resulting in potentially clinically unnecessary diagnostic suggestions (17).

Current research provides preliminary insights into the potential causal relationship between mobile health applications and patients’ overutilization of medical services. For instance, a study by Carroll et al. (18) highlighted that the electrocardiogram feature of the Apple Watch has turned a segment of the population into proactive healthcare consumers, which may lead to repeated self-screening and overutilization of medical services (19). Additionally, other research suggests that medical information overload can induce cyberchondria, indirectly affecting people’s vaccination behaviors (20), and during pandemics, an overload of online medical information may prompt self-isolation behaviors (21). A study focusing on China indicated that excessive use of tracking mobile health applications might enhance individuals’ sense of responsibility and awareness of consequences, thereby aligning with epidemic prevention measures (22). Hence, when mobile health applications are predominantly informational (23), online medical information can trigger cyberchondria, leading to various health-related behaviors. Thus, this indicates that mobile health might become a new factor contributing to the overutilization of medical services. However, there is a research gap in this area.

To address these questions, we will base our study on the Health Belief Model to construct research hypotheses and a research model, conducting a survey among users of mobile health apps in China. The main objective of this study was to investigate the relationship between information overload in mobile health (mHealth) applications and overutilization of medical services. Specifically, this study aims to: (1) Examine how information overload affects users’ perceptions of severity, susceptibility, treatment benefits, barriers, self-efficacy, and action cues; (2) Based on the Health Belief Model (HBM), the psychological mechanism of users’ overuse of medical services in the face of excessive medical information was studied; (3) Provide actionable recommendations for mobile health app developers, healthcare providers, and policymakers to mitigate the adverse effects of information overload and encourage smarter use of healthcare resources.

This study has multiple important implications. Firstly, although the widespread adoption of mobile health applications has provided users with unprecedented access to medical information, research on the impact of information overload on healthcare decision-making is still insufficient. This study fills the research gap by exploring how information overload in mobile health applications affects users’ health perception and their tendency to overuse medical services. Secondly, this study introduces the Health Belief Model (HBM) into the field of digital health, thus expanding the theoretical understanding of the mechanisms by which cognitive and psychological factors (such as perceived severity, susceptibility, and self-efficacy) are affected in digital information environments. Finally, this study provides practical recommendations for mobile health application developers, healthcare providers, and policymakers to design more user-friendly interfaces to reduce information overload and help users make more informed and rational healthcare decisions. Additionally, these findings can serve as a basis for establishing standards for the quality and delivery of medical information on digital platforms, ultimately easing unnecessary pressure on the healthcare system and promoting public health.

## 2 Literature review and hypothesis

### 2.1 The health beliefs model

The Health Belief Model (HBM) was initially developed by social psychologists at the U.S. Public Health Service in the 1950s, aimed at explaining the psychological motivations behind individuals’ participation in disease prevention and detection behaviors. Becker et al. (24) further elaborated that the HBM comprises four core components: the perceptions of susceptibility to and severity of a specific health threat, as well as the perceived benefits and barriers to taking preventive or curative actions. Additionally, Rosenstock (25) introduced the construct of “cues to action,” emphasizing that external or internal stimuli can trigger individuals’ awareness of potential adverse health consequences, thereby motivating them to act. Building on this, Jeong & Ham (26) noted that these cues could stem from internal perceptions (such as physical symptoms) or external factors (such as social interactions and media influence). Over time, research on the HBM has deepened, with the model being expanded to include more variables that influence individual health behaviors, such as self-efficacy, motivational factors, and personality traits, all playing significant roles in individuals’ health behavior decisions (27). The HBM has become one of the mainstream theoretical frameworks for explaining and predicting individual health behaviors, not only elucidating people’s participation in disease prevention and detection but also providing a theoretical basis for health behavior interventions (27).

HBM’s empirical research shows that the main feature of this model is that it can effectively predict and explain the behavior of individuals in different health fields, such as preventive health behaviors (28), promoting health behaviors in patients with chronic kidney disease (29), HIV/AIDS prevention behaviors (30), and the health beliefs and promotive behaviors of middle-aged women (31). Carpenter (32) provided some of the most compelling evidence for the HBM’s predictive power regarding health-related behaviors through a meta-analysis encompassing 18 studies with 2,702 participants.

Therefore, using the Health belief Model (HBM) to study the relationship between information overload and overuse of health services has several unique advantages. First, HBM is able to explain how individuals make health behavior decisions based on their perceptions of susceptibility and severity of disease, as well as cognitive benefits and barriers to preventive or therapeutic measures. Second, HBM can integrate the two important factors of self-efficacy and action prompting. This mechanism could help explain why users tend to adopt excessive health behaviors when faced with a constant barrage of information from mobile health apps. Through this theoretical lens, we can gain deeper insights into the psychological and social drivers behind the overutilization of medical services, providing a theoretical basis for the development of corresponding intervention measures.

### 2.2 Information overload

Information overload refers to a scenario where the demand for information processing surpasses an individual’s actual capacity to process information. Time is a critical dimension in assessing the imbalance between information processing demands and capabilities, that is, whether the capacity to process information within a given time aligns with the volume of information that needs processing (33). Information overload is commonly encountered during information retrieval, analysis, and decision-making processes (34) and can lead to difficulties in synthesizing information, increased anxiety and stress, ultimately affecting the quality and efficiency of decision-making (35).

In the healthcare sector, the issue of information overload has garnered considerable attention. Information overload in healthcare poses significant challenges to patients’ health behaviors. Firstly, the rapid evolution of medical information may leave patients unable to keep up with the latest developments, leading to an information asymmetry between patients and healthcare providers. Secondly, patients may feel overwhelmed during communications with doctors, struggling to fully comprehend medical advice and diagnoses (36). For instance, information overload has been identified as a significant predictor of the lack of knowledge about oral anticoagulants, which can result in patients’ non-adherence to oral anticoagulant therapy plans (37). Furthermore, when faced with an abundance of information, some patients might attempt self-diagnosis and treatment, searching online for symptom or medication information, which could lead to misunderstandings and inappropriate treatment actions, increasing health risks. For example, information overload has been found to impact patients’ willingness to undergo regular health check-ups (38), and affect the intention to receive vaccinations during a pandemic (20). Lastly, healthcare information overload can lead to psychological issues such as anxiety and distress in patients (39). When confronted with contradictory or inaccurate information from the internet or media, patients’ concerns increase, and the uncertainty about diseases and treatments intensifies. Studies confirm that people exposed to online information are more prone to experience information overload, which can lead to cyberchondria and an increased perception of the severity of diseases (21).

In the healthcare sector, there is a potential link between information overload and the overutilization of medical services. Amidst the vast amount of information patients encounter, some may be inaccurate, inconsistent, or challenging to comprehend. When experiencing information overload, patients might lean toward seeking excessive medical interventions, such as unnecessary tests or treatments, possibly due to misunderstandings of medical information or an exaggerated focus on health risks. Furthermore, information overload can heighten patients’ expectations regarding diagnosis and treatment, leading to unnecessary demands or interventions with their healthcare providers, based on the expectations formed from the information they have encountered.

According to the Pew Research Center, health-related information is among the most searched-for topics on the internet (40). Online health information searches have replaced traditional face-to-face medical consultations, reducing the frequency of traditional medical visits. However, with the rapid growth of mobile health applications and health information, patients find it challenging to efficiently locate accurate information, leading to a health information processing burden that exceeds individual capacity and triggers information overload (39). While the swift development of mobile health brings value to public health and simplifies the connection between patients and healthcare providers, it also carries the potential risk of exposing patients to an overwhelming amount of unfiltered or complex information. Therefore, examining the relationship between information overload in mobile health and the overutilization of medical services is particularly crucial.

### 2.3 Research model

In this study, we employ the Health Belief Model (HBM) as the theoretical framework to explore patients’ health behaviors in the context of mobile health applications. The HBM posits that an individual’s health decision-making process is influenced by their perceived severity of and susceptibility to a disease, perceived effectiveness of treatment or preventative measures, perceived barriers to performing specific health behaviors, self-efficacy, as well as personality and motivational factors (27). Building on this, our research incorporates perceived severity, perceived susceptibility, perceived benefits of treatment, perceived barriers to treatment, self-efficacy, and intention to act into the model to predict patients’ overutilization of medical services when using mobile health applications.

**Figure 1:**
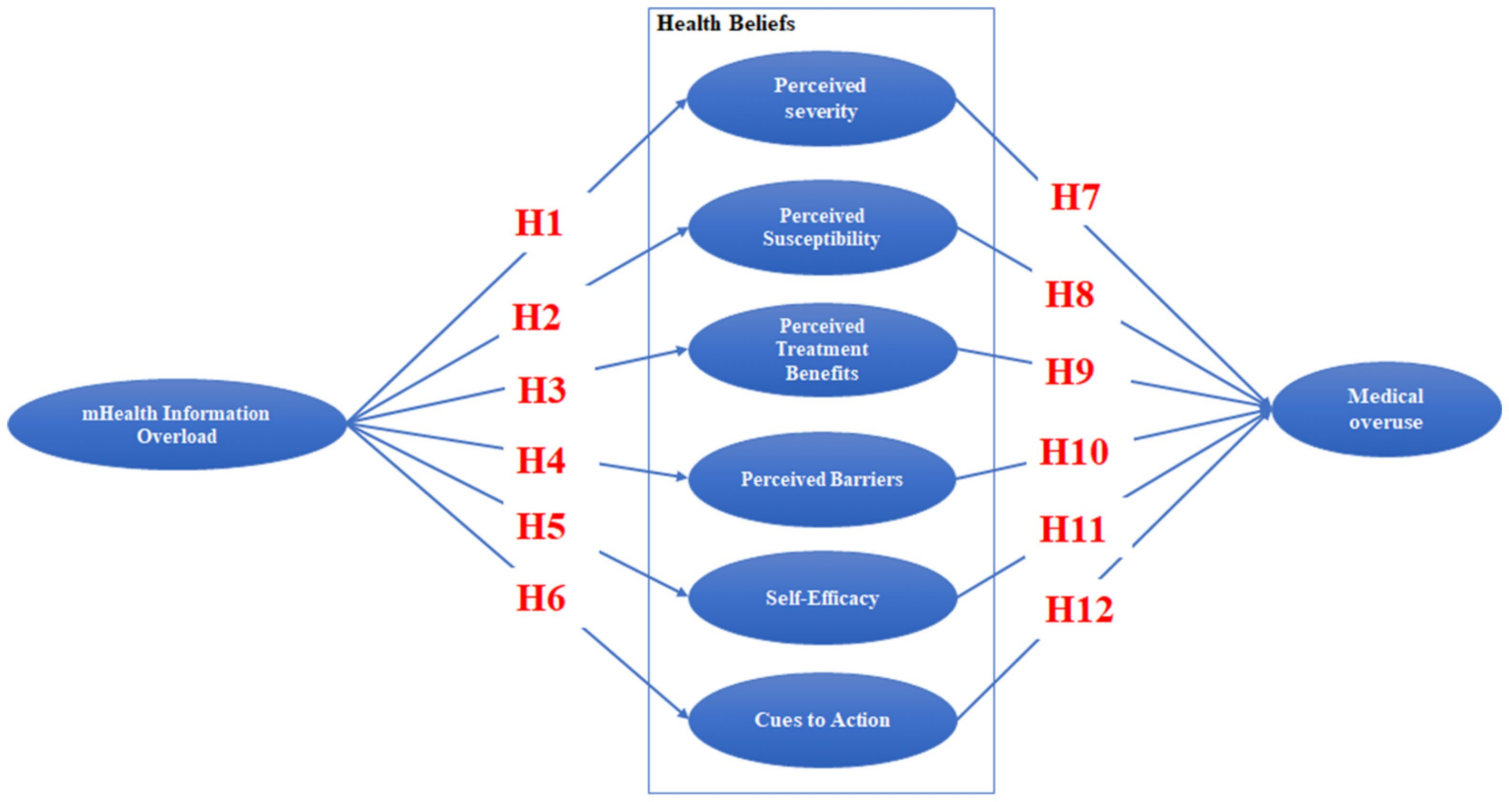
Research model.

Mobile health applications, being a primary source of modern medical information, provide two main functions: one is the search for medical knowledge, where the app provides relevant information; the other is online medical consultation, allowing users to interact with online doctors. This study aims to examine how information overload in mobile health affects the six key factors in the HBM to predict patients’ behavior regarding the overutilization of medical services. The research model for this study is illustrated in Figure 1.

### 2.4 Hypothetical development

Perceived severity is defined as an individual’s assessment of the seriousness of their health condition and its potential consequences, reflecting the individual’s perception of the severity of their current and future health status. In the digital health environment, users are exposed to a vast amount of health information, which can trigger information overload. This phenomenon can affect users’ perception of the severity of health threats. Previous research indicates a positive correlation between information overload and perceived severity (21). Particularly in mobile health applications, users are confronted with an abundance of health warnings and risk data that may exceed their information processing capabilities, increasing health-related anxiety. Prolonged exposure can lead to excessive vigilance, self-diagnosis, and even symptoms such as heightened anxiety, fatigue, and insomnia in users (41). Information overload in the mHealth environment may lead users to erroneously believe their health condition is extremely severe, a perceptional imbalance due to inadequate information processing capacity rather than a rational assessment based on objective medical evidence. Hence, this study proposes the following hypothesis:

*H1*: In the use of mobile health apps, information overload has a positive impact on users’ perceived severity of health.

In the digital health era, frequently searching for medical information on the internet can exacerbate illness anxiety, a phenomenon referred to as “cyberchondria,” which is closely associated with excessive searching of health information online (42). With the widespread adoption of mobile health applications and online health platforms, the amount of medical information users are exposed to via mobile devices has significantly increased. While this enhances user awareness of health issues, it can also potentially lead to information processing overload. Information overload may make it difficult for users to discern the accuracy and reliability of information, thereby increasing unnecessary concerns about illness. For instance, among men who have sex with men/bisexual men who are at high risk but not infected with HIV, continuous attention to the threat of HIV infection has led to depression and anxiety (43). Furthermore, due to personalized recommendation algorithms, users might be inundated with a large volume of similar information, isolating them from other sources of information and further exacerbating anxiety and panic. The personalized health information provided by mobile health apps could intensify users’ concerns about illnesses, especially when the apps offer detailed information on disease progression, real-time health metrics feedback, or personalized health predictions, making users feel more sensitive and vulnerable to health issues. Therefore, this study proposes the following hypothesis:

*H2*: In the use of mobile health apps, information overload has a positive impact on users’ perceived susceptibility to health issues.

Information overload has a significant impact on individuals’ perceptions and decision-making across various domains. In the field of public administration, Cao et al. (35) demonstrated that information overload has a positive impact on individuals’ perception of policy benefits. Similarly, in the educational sector, Chen et al. (44) found that information overload could lead to inefficient learning and deplete students’ cognitive resources. In the realm of public health, Hoffmann & Del Mar (45) observed that most patients tend to overestimate the benefits of medical interventions due to unrealistic expectations while underestimating potential harms.

Particularly in the context of using mobile health apps, the influx of a large volume of medical information can lead to information overload during medical decision-making, especially when processing information about the benefits of treatment methods (46). Information overload can exacerbate cognitive biases towards the perceived advantages of treatment options, prompting users to selectively focus on information that supports the benefits of certain treatments, thus reinforcing their perception of these benefits. The overestimation of treatment benefits caused by information overload could lead to an increase in placebo effects, enhancing individuals’ confidence in the effectiveness of treatments (47), thereby potentially impacting health outcomes. Therefore, this study proposes the following hypothesis:

*H3*: In the use of mobile health apps, information overload has a positive impact on users’ perceived benefits of treatment.

Information overload is considered one of the main factors hindering individuals from understanding their health conditions and taking appropriate medical actions (48). Information overload occurs when the volume of information exceeds an individual’s processing capacity, requiring users to discern between beneficial, superfluous, erroneous, and meaningless information (49). Users are constrained in evaluating the accuracy and reliability of information, which significantly impacts the quality of decision-making. Information overload can lead to reduced decision accuracy (50), cause information to be misinterpreted or over-interpreted, provoke unnecessary anxiety (51), and potentially mislead users into making incorrect self-diagnoses or inappropriate treatment choices, thereby enhancing perceived treatment barriers.

The research by Pchelina et al. (41) corroborates the negative impacts of information overload on individuals’ health and sleep, potentially leading to psychological states such as anxiety and depression. In the mobile health context, information overload influences users’ perception of treatment barriers. Faced with an abundance of information, users may experience cognitive confusion and uncertainty, hindering their ability to make rational health and treatment decisions. This uncertainty can lead to hesitancy in seeking medical help, becoming an obstacle to effective treatment and recovery. Treatment barriers may encompass aspects such as the cost, time, and accessibility of specific treatments. Information overload intensifies patients’ confusion and stress regarding the treatment process, increasing perceived barriers. When dealing with a large volume of complex information, especially information closely related to their health status, patients may feel overwhelmed, thereby heightening the sense of uncertainty around the treatment process, as well as psychological and practical barriers. Therefore, this study proposes the following hypothesis:

*H4*: In the use of mobile health apps, information overload has a positive impact on users’ perceived treatment barriers.

Self-efficacy refers to an individual’s belief in their capability to execute specific behaviors to achieve desired outcomes. In the digital health environment, accessing a large amount of medical information can impact patients’ self-efficacy. This change in self-efficacy might stem from patients’ perception that they have acquired more health information, enabling them to make more informed personal health decisions. For instance, Wiljer et al. found that breast cancer patients’ access to electronic health records was positively correlated with their perception of self-efficacy (52). This impact could be influenced by various factors, including the individual’s health literacy, the clarity and relevance of the information received, and the individual’s prior experience in health decision-making.

Positive experiences can enhance an individual’s sense of efficacy, while negative experiences might lead to its reduction. However, information overload can trigger heuristic thinking in users (53), affecting the efficacy of experiences and possibly leading to individuals overestimating their abilities (54). This overestimation can result in patients developing blind confidence, which might ultimately lead to decision-making errors. In the mobile health context, users are confronted with a vast amount of information and may not be able to process this information effectively, potentially misleading their evaluation of their own capacity to handle health information and make health decisions. Therefore, this study proposes the following hypothesis: Therefore, this study proposes the following hypothesis:

*H5*: In the use of mobile health apps, information overload has a positive impact on users’ self-efficacy.

In the digital health environment, accessing medical information is a double-edged sword for patients. On one hand, it empowers patients to effectively search for online health information, potentially increasing action cues, thereby promoting the occurrence of health behaviors (48). On the other hand, information overload can influence users’ behavioral patterns through psychological mechanisms. For instance, research by Honora et al. (20) shows that information overload can reduce vaccination intentions by increasing skepticism about vaccines. Similarly, Laato et al. (21) found that information overload leads to cyberchondria, triggering panic-buying behaviors. This dichotomy highlights the complex interplay between information access and user outcomes in the realm of digital health.

The relationship between information overload and the effectiveness of action cues is complex and varies depending on situational factors. While information overload can sometimes lead to confusion and hinder decision-making, it may enhance the response to action cues when users perceive the information to be structured, offering clear guidance and motivation. In mobile health applications, the phenomenon of information overload is widespread, with patients often confronting a plethora of health advice, preventive measures, and treatment options. This not only imposes a cognitive burden but also provides opportunities to motivate changes in health behaviors. Information overload might inspire patients to adopt more proactive information search and processing strategies, identifying more action cues. Therefore, this study proposes the following hypothesis:

*H6*: In the use of mobile health apps, information overload has a positive impact on users’ perception of action cues.

Perceived severity, defined as an individual’s psychological assessment of the seriousness of a health threat, is a core factor that influences medical decisions and behaviors. For instance, studies during the COVID-19 pandemic found that high perceived severity of infection prompted patients to be more likely to engage in self-isolation behaviors (21, 55). There is empirical support that perceived severity is positively correlated with the overutilization of medical services. Driven by concerns about the potential consequences of diseases, patients may tend to opt for more diagnostic tests and treatment measures, even though these additional medical activities might not be necessary objectively (56). This highlights the impact of perceived severity on health-related decision-making and actions, potentially leading to an increase in healthcare resource utilization based on subjective health threat assessments rather than objective medical necessity.

In the digital health environment, particularly through mobile health applications, patients are exposed to an abundance of information regarding disease descriptions, treatment options, and risk factors. When patients have a high perceived severity of disease, this could exacerbate their concerns about health issues, making them more receptive to additional treatment measures. Consequently, they might adopt more aggressive medical behaviors, increasing the risk of overutilization of medical services. Therefore, this study proposes the following hypothesis:

*H7*: The perceived severity of health by patients has a positive impact on their willingness to overuse medical services.

Perceived susceptibility, defined as the degree to which an individual believes they are susceptible to a particular disease or health issue, is influenced by various factors, including personal or family medical history, the volume of health information encountered, and media coverage (57, 58). Higher perceived susceptibility can motivate individuals to pay closer attention to health risks and take steps to mitigate those risks (59, 60), leading to increased focus on threatening information (61). This heightened focus can motivate them to take proactive measures against health threats. However, it may also trigger behaviors associated with the overutilization of medical services, as patients’ increased anxiety and concern drive them to seek out more health interventions, potentially exceeding what is medically necessary.

In the mobile health app environment, patients with high perceived susceptibility may use the app more frequently to obtain information about symptoms, preventive measures, and treatment methods. This frequent exposure to information might exacerbate their concerns, prompting them to seek additional medical interventions to alleviate anxiety. The self-diagnosis and health monitoring features provided by mobile health apps could be overly relied upon by highly susceptible patients, leading to unnecessary anxiety and the behavior of overutilizing medical services. Moreover, the online consultation and appointment scheduling features of these apps might be frequently used by highly susceptible patients, increasing unnecessary doctor visits and medical examinations, thereby intensifying the risk of medical service overutilization. Therefore, this study proposes the following hypothesis:

*H8*: The perceived susceptibility to diseases by patients has a significant positive impact on their willingness to overuse medical services.

The overutilization of healthcare services is indeed a complex issue, and this is mirrored in the design of mobile health (mHealth) applications. Developers often include an extensive amount of information and offer multiple solutions within these apps to demonstrate their comprehensiveness (62, 63). However, these applications may lack credibility, applicability, personalization, and accessibility, rendering the content they provide not always able to meet the specific needs or preferences of individual patients (64). Moreover, patients’ unrealistic expectations regarding treatment outcomes are a significant factor leading to the overuse of healthcare services (45). Patients may erroneously believe that more or costlier treatments yield better outcomes, thus seeking or consenting to unnecessary treatments (5). Uncertainty in the diagnostic process is another key factor, as medicine is not always an exact science (65). This uncertainty can leave patients who rely on information from mobile health applications feeling confused. To eliminate the potential risks of missed or incorrect diagnoses, patients may lean towards seeking additional examinations and treatments, even when these measures are not necessary in some cases. Under these interrelated factors, patients’ positive perceptions of treatment benefits may further promote the occurrence of healthcare service overutilization. Patients’ beliefs and expectations largely determine their acceptance of treatments (66). When patients firmly believe that a certain treatment or diagnostic procedure will bring significant benefits, they are more likely to consent to these treatments, even if they are not medically strictly necessary. Therefore, this study proposes the following hypothesis:

*H9*: Patients’ perceived benefits of treatment has a positive impact on their willingness to overuse healthcare services.

Treatment barriers refer to the various difficulties individuals encounter when accessing specific treatments, such as cost, time, accessibility, and other factors. Typically, perceived treatment barriers would deter patients from pursuing or continuing treatment. However, in the context of mobile healthcare, this relationship exhibits different characteristics. In this context, patients’ perception of treatment barriers may positively correlate with their tendency to overuse healthcare services. This phenomenon can be explained through psychological and socioeconomic mechanisms. When patients face perceived obstacles, they may seek faster or more significant treatment outcomes in order to ease health concerns. From a behavioral economics perspective, patients facing higher perceived barriers might prefer actions that offer immediate relief from anxiety or symptoms, especially when experiencing severe discomfort. They may opt for quicker symptom relief methods rather than longer-term but milder treatment options (67). Additionally, socio-psychological factors also play a crucial role (68). For instance, patients may pursue treatments perceived to provide quick solutions due to social and cultural pressures. In physician-patient communication, the doctor’s advice is often seen as authoritative guidance. When faced with high perceived treatment barriers, patients may be more inclined to unconditionally accept doctors’ treatment suggestions, even if these recommendations could lead to the overutilization of healthcare services.

While mobile health applications offer convenient access to health information and advice, reducing physical barriers, they may concurrently elevate cognitive and emotional obstacles for patients, such as excessive health anxiety and unrealistic expectations for treatment outcomes. When encountering significant perceived treatment barriers, patients might be more inclined to seek additional medical interventions, like unnecessary diagnostic tests or treatments. Concerns regarding uncertainties or side effects could also lead patients to pursue more medical opinions or alternative therapies, thereby heightening the risk of healthcare service overutilization. Accordingly, this study puts forth the following hypothesis:

*H10*: Patients’ perceived treatment barriers has a positive impact on their willingness to overuse healthcare services.

Self-efficacy, the belief in one’s capabilities to organize and execute the courses of action required to manage prospective health behaviors and decisions, is a significant psychological factor influencing health behavior (69). In the context of mobile health, users receive health information and feedback through applications, an interaction that can enhance their sense of self-efficacy, thereby motivating them to more actively participate in self-management and medical decision-making processes. For instance, studies have shown that heart failure patients using the HeartMapp mobile application experienced enhanced confidence and user experience (70), suggesting that mobile applications can encourage individuals to actively engage in self-management, thereby boosting their self-efficacy to achieve desired health outcomes.

Self-efficacy and response efficacy play pivotal roles in individuals’ acceptance of mobile health services, influencing their perceptions of the usability and effectiveness of applications (71). Patients with high self-efficacy are more confident, a trait that propels them to use mobile health applications more frequently and to proactively explore medical options, including non-essential treatments. Believing in their ability to manage complex medical information, these patients tend to adopt proactive healthcare measures, such as excessive monitoring, diagnostics, and interventions, which may lead to an overreliance on medical interventions. Additionally, patients with high self-efficacy may hold optimistic expectations about treatment outcomes (72). They might overly focus on the benefits described in treatment plans while underestimating the likelihood of side effects (73), thereby overlooking the potential risks associated with medical interventions. Therefore, this study proposes the following hypothesis:

*H11*: Patients’ self-efficacy has a positive impact on their willingness to overuse medical services.

In the Health Belief Model, “cues to action” are internal or external factors that motivate individuals to change their behavior. These cues can come from various channels, including public health information, doctors’ advice, and more. An increase in cues to action is believed to potentially enhance the likelihood that individuals will adopt healthy behaviors. For instance, one study demonstrated that reminder postcards developed based on elements of the Health Belief Model significantly increased vaccination rates (74). However, in the context of mobile health applications, cues to action that are unfiltered or lack appropriate medical guidance may increase the risk of overutilizing medical services. This is because patients might interpret each cue to action as a signal that immediate medical intervention is needed, prompting them to excessively use medical services.

Existing empirical research has begun to focus on such relationships. Patients who receive an abundance of cues to action, such as widespread health screening campaigns, excessive attention to potential health risks, and frequent recommendations about treatment from family and friends, are more likely to request or agree to additional medical examinations and treatments (75). Mobile health applications provide an abundance of cues to action through health notifications, reminders, and suggestions. These excessive prompts may lead to patients’ overreaction and exacerbate the risk of overutilizing medical services. Frequent cues can heighten patients’ concerns about health issues, prompting them to seek additional diagnostics or treatments to alleviate anxiety. This anxiety can lead patients to have exaggerated expectations of the need for and effectiveness of treatment without sufficient evidence. Therefore, this study proposes the following hypothesis:

*H12*: Patients’ perceived cues to action have a positive impact on their willingness to overuse medical services.

## 3 Methods

### 3.1 Measurement, sampling and data collection

This study has designed a questionnaire aimed at assessing the relationship between information overload in mHealth applications and users’ tendency to overuse medical services. The items in the questionnaire employ a 5-point Likert scale, with all entries being based on existing research and suitably adjusted and adapted for the context of this study. To ensure the completeness of the questionnaire, all questions are set as mandatory, and submissions of incompletely answered questionnaires will not be accepted. In recent years, digital data collection methods have become increasingly popular due to their high efficiency and wide coverage. These methods enable researchers to access diverse populations and efficiently collect large amounts of data, with significant advantages especially in fields such as social and management sciences (76). From the respondents’ perspective, Monday and Gever found through their research that online surveys have a 16% higher response rate than in-person surveys, with the majority of respondents preferring to participate via digital platforms (77). From the point of view of researchers, Gever et al. have demonstrated that digital data collection methods have the advantages of collecting large-scale and diverse data, cost effectiveness, and timely collection, which are well suited for contemporary research (78, 79). Therefore, this study takes the online survey tool Questionnaire Star to survey users of digital media platforms of medical treatment.

Following the questionnaire’s development, experts in the field of public health were invited to review the survey instrument to ensure its content validity and relevance. Moreover, 60 undergraduate students participated in a pilot test of the questionnaire to verify its comprehensibility and logical structure. The constructs in the questionnaire were specifically adapted from the following sources: the mobile health information overload section was adapted from Cao et al. (35); perceived severity (PSEV) and perceived susceptibility (PSUS) were adapted from Walrave et al. (80); perceived treatment benefits (PTB) from Liu et al. (81); perceived treatment barriers (PBA) from Adiyoso et al. (82); self-efficacy (SE) from Wu et al. (83); and cues to action (CTA) Arabyat et al. (84). Given the lack of empirically validated scales for overuse of medical services in the medical field, this study referred to Ding et al. (85) scale on smartphone overuse and, combined with the pilot test results, developed a scale for medical service overuse suitable for the context of this study. The complete scale is included in Appendix A of the document.

Before conducting the survey, this study received approval from the university’s ethics committee to ensure the ethical conduct of the research. All participants were clearly informed about the following aspects before completing the questionnaire: the anonymity of the survey, the content and purpose of the research, the voluntary nature of their participation, the absence of personal privacy information in the survey, and the incentives provided upon completion of the questionnaire.

**Figure 2:**
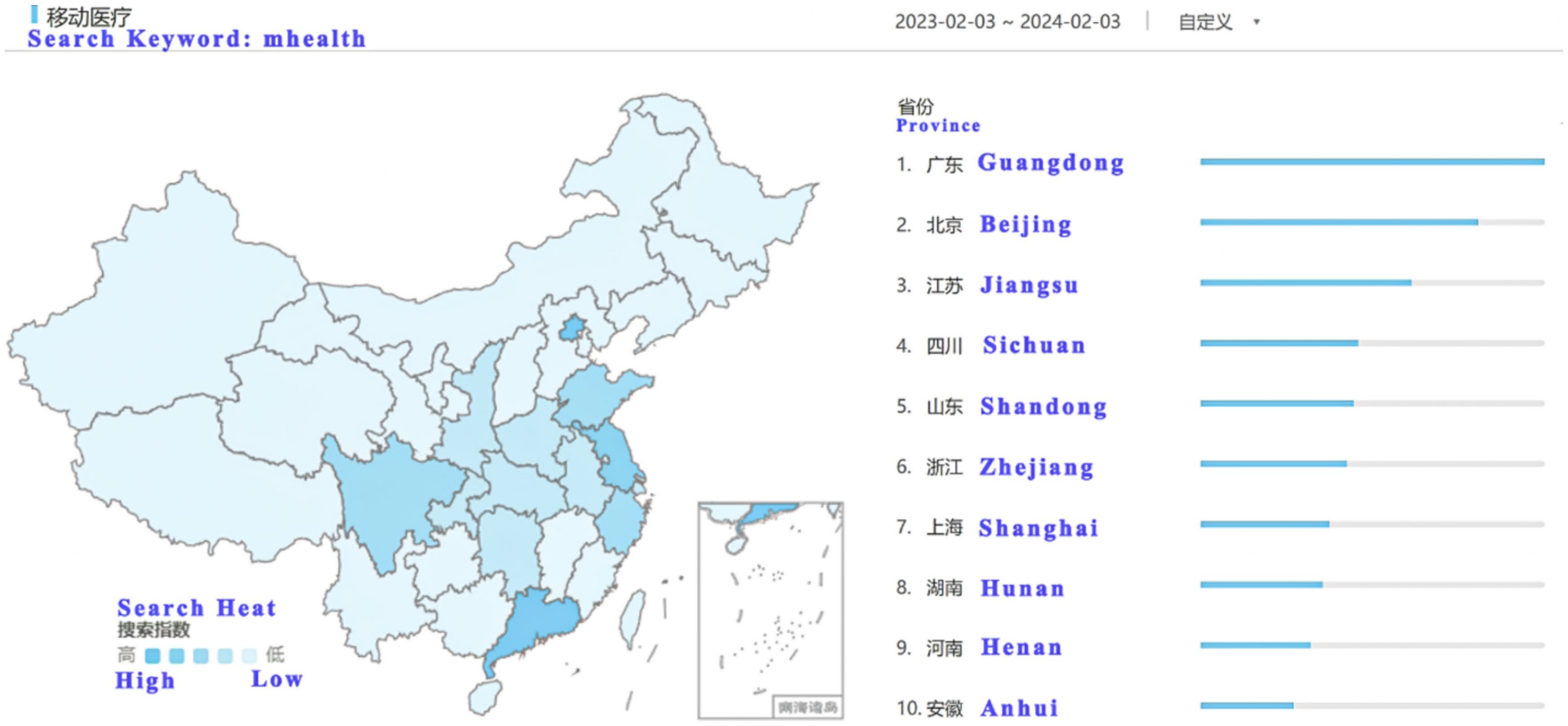
Baidu index.

Baidu Index is a data analysis platform based on Baidu’s massive Internet user behavior data, which is widely used in market research, brand analysis, trend prediction and other fields. It reflects the public’s attention to a specific topic by counting the frequency of users’ keyword searches in Baidu’s search engine (86). As Baidu is one of the most commonly used search engines by Chinese users (87), the search data of Baidu Index has strong representativeness and authority, and has high reference value. Based on data from Baidu Index^1^, there was a high level of interest in digital healthcare in Guangdong Province. In addition, the purpose of this study is to explore the user behavior characteristics in areas with high attention and understand the relationship between information overload and overuse of medical services, rather than the national general behavior. Therefore, the target population for this study was identified as mobile health app users in Guangdong Province. Using a random sampling method, questionnaire was distributed from 2024.2.4 to 2.20. A total of 1,494 responses to the questionnaire were collected, of which 1,137 were valid responses, resulting in a valid response rate of 76.1% (Figure 2).

### 3.2 Demographic details of the survey respondents

Table 1 presents the demographic information of the 1,137 respondents. The proportion of female respondents (*N* = 693, 60.95%) is higher than that of male respondents (*N* = 444, 39.05%). The age of the respondents is predominantly between 30 to 49 years old (*N* = 745, 65.52%). The monthly income of most respondents falls between 9,000 RMB to 11,999 RMB (*N* = 543, 47.76%).

**Table 1:** Demographic characteristics of respondents.

Demographic characteristics	Item	*N*	Percentage %
Gender	Female	693	60.95%
	Male	444	39.05%
Age	20–29	90	7.92%
	30–39	222	19.52%
	40–49	523	46.00%
	>50	302	26.56%
Income (RMB/Month)	3,000–5,999RMB	60	5.28%
	6,000–8,999RMB	138	12.14%
	9,000–11,999RMB	543	47.76%
	12,000-14,999RMB	278	24.45%
	>15,000RMB	118	10.37%

In the present investigation, the proposed model incorporated eight distinct variables. Subsequently, an assessment of multivariate normality was executed on the amassed data, utilizing an online computational tool^2^ to ascertain the data’s distribution profile. The findings delineated Mardia’s multivariate skewness (*β* = 28.623, *p* > 0.05) and kurtosis (*β* = 1051.143, *p* < 0.001), indicating a deviation from multivariate normality (88). This investigation is characterized as exploratory in nature. In essence, the applicability of this study extends to data analyses employing Partial Least Squares Structural Equation Modeling (PLS-SEM).

### 3.3 Data analytical tool

In this study, we examine latent psychological variables that cannot be directly measured. To validate the proposed hypotheses, the study employed Structural Equation Modeling (SEM) to analyze the collected data.

Structural Equation Modeling (SEM) is broadly categorized into two types: Covariance-Based Structural Equation Modeling (CB-SEM) and Partial Least Squares Structural Equation Modeling (PLS-SEM). PLS-SEM, as a second-generation multivariate data analysis method, is primarily suited for exploratory theoretical research, allowing for the analysis while ensuring the integrity of all relationships between independent and dependent variables (89).

PLS-SEM offers several significant advantages over CB-SEM: (1) PLS-SEM is more suitable for complex models involving more than six variables (90). (2) PLS-SEM can effectively handle small sample data (90). (3) PLS-SEM is applicable to data that are not normally distributed (90). Given these factors, PLS-SEM has more adaptability than CB-SEM in the theory development stage and has been proven to effectively replace CB-SEM in most social science research contexts (90).

Secondly, Asogwa et al. ‘s research shows that digital software is significantly superior to manual methods in terms of analytical efficiency, subject recognition, and visualization techniques, especially when dealing with complex data sets (91). Therefore, in this study, in order to improve the efficiency of qualitative data analysis, we choose PLS-SEM and its supporting digital software SmartPLS 4.0 for data analysis.

### 3.4 Measurement deviation

Common method bias is a prevalent issue in questionnaire surveys. Harman’s single factor analysis, introduced by Harman in 1976, is widely employed in social science research to detect common method bias (92). This method suggests extracting a single factor, and if the variance is less than 40%, it indicates minimal influence of common method bias on the survey data (92). The Harman analysis conducted in this study revealed a proportion of 30.49% for the extracted variables (below 40%).

Furthermore, we conducted a Full-Variance Inflation Factor (Full-VIF) Test on the data to further examine common method bias. Research suggests that applying the Full-VIF Test to all variables in the model, including dummy variables, can help detect potential common method bias. If the Variance Inflation Factor (VIF) exceeds 3.3, it indicates a possible influence of common method bias on the model. However, if all VIF values obtained from the Full-VIF Test are equal to or below 3.3, it can be inferred that the model is not significantly affected by common method bias (93). This method has been widely applied across various research domains (88). The test results of this study found that all VIF values were below 3.3. Considering the results of both methods used to test for common method bias, it can be concluded that common method bias is not a significant concern in this study.

A paired *t*-test was employed to scrutinize the potential nonresponse bias within the demographic data of the initial and concluding 25 participants. The outcome revealed no statistically significant disparities, thereby indicating that nonresponse bias did not pose a substantive issue within the context of this investigation.

## 4 Results

### 4.1 Measurement model

The quality of the model was assessed through the evaluation of composite reliability (CR), average variance extracted (AVE), discriminant validity, and outer loading. As shown in Table 2, the composite reliability and Cronbach’s alpha of each variable exceed 0.7, indicating satisfactory internal consistency of the data in this study. Additionally, the AVE values of each variable surpass 0.5, with outer loadings exceeding 0.7, confirming the acceptable convergent validity in this study (90).

**Table 2:** Reliability and validity of constructs.

Latent variable	Item	Loading	Mean (SD)	Cronbach’s *α*	CR	AVE	*R* ^2^
MHIO	MHIO1	0.868	3.196(0.565)	0.860	0.902	0.696	
	MHIO2	0.835					
	MHIO3	0.822					
	MHIO4	0.812					
PSEV	PSEV1	0.753	2.967(0.985)	0.819	0.881	0.651	0.041
	PSEV2	0.849					
	PSEV3	0.872					
	PSEV4	0.745					
PSUS	PSUS1	0.795	3.006(1.002)	0.833	0.889	0.668	0.049
	PSUS2	0.873					
	PSUS3	0.862					
	PSUS4	0.731					
PTB	PTB1	0.709	3.008(0.992)	0.824	0.882	0.653	0.006
	PTB2	0.892					
	PTB3	0.873					
	PTB4	0.743					
PBA	PBA1	0.781	3.033(1.002)	0.837	0.892	0.674	0.037
	PBA2	0.860					
	PBA3	0.874					
	PBA4	0.764					
SE	SE1	0.799	2.999(1.036)	0.848	0.898	0.688	0.023
	SE2	0.903					
	SE3	0.845					
	SE4	0.765					
CTA	CTA1	0.797	3.006(0.993)	0.829	0.887	0.664	0.022
	CTA2	0.882					
	CTA3	0.860					
	CTA4	0.709					
MOU	MOU1	0.852	3.428(0.548)	0.829	0.878	0.644	0.221
	MOU2	0.787					
	MOU3	0.790					
	MOU4	0.779					

MHIO, mHealth information overload; PSEV, perceived severity; PSUS, perceived susceptibility; PTB, perceived treatment benefits; PBA, perceived barriers; SE, self-efficacy; CTA, cues to action; MOU, overuse of health services.

Table 3 presents the results of Fornell and Larcker’s Test and the Heterotrait-Monotrait ratio (HTMT) Test, used to assess discriminant validity. The HTMT values between variables are below the threshold of 0.85, and the square root of the AVE for each variable exceeds its correlation with other variables, as suggested by (90). A comprehensive analysis indicates that this study exhibits good levels of composite reliability, convergent validity, and discriminant validity.

**Table 3:** Discriminant validity.

Fornell-Larcker criterion								
	CTA	MHIO	MOU	PBA	PSEV	PSUS	PTB	SE
CTA	0.815							
MHIO	0.149	0.834						
MOU	0.188	0.276	0.802					
PBA	0.021	0.191	0.229	0.821				
PSEV	−0.032	0.202	0.220	−0.042	0.807			
PSUS	−0.001	0.222	0.230	0.004	−0.040	0.817		
PTB	−0.071	0.077	0.042	−0.015	0.080	0.005	0.808	
SE	0.022	−0.152	−0.147	0.002	0.026	−0.061	0.049	0.830

Heterotrait-Monotrait Ratio								
	CTA	MHIO	MOU	PBA	PSEV	PSUS	PTB	SE
CTA								
MHIO	0.159							
MOU	0.198	0.263						
PBA	0.036	0.213	0.251					
PSEV	0.061	0.228	0.237	0.054				
PSUS	0.033	0.242	0.242	0.042	0.053			
PTB	0.091	0.086	0.048	0.034	0.102	0.027		
SE	0.047	0.154	0.145	0.031	0.041	0.074	0.065	

MHIO, mHealth information overload; PSEV, perceived severity; PSUS, perceived susceptibility; PTB, perceived treatment benefits; PBA, perceived barriers; SE, self-efficacy; CTA, cues to action; MOU, overuse of health services.

### 4.2 Structural model

Before conducting structural model measurement, we assessed collinearity, and the VIF values for each variable were below 3. Hence, collinearity is not a significant concern in this study. After ensuring the reliability and validity of the model, we used the structural model to validate the hypotheses. The research results show that information overload caused by the use of mobile medical software has significant positive correlation with perceived severity, perceived susceptibility, perceived treatment benefits, perceived cure obstacles, self-efficacy and action cues, and H1, H2, H3, H4, H5, and H6 are all supported. Perceived severity and perceived susceptibility were positively correlated with overuse of medical services, that is, H7 and H8 were supported. However, the perceived benefit of treatment showed no significant effect on overuse of medical services, and H9 was not supported. Finally, perceived healing barriers, self-efficacy, and action cues were positively correlated with medical overuse, that is, H10, H11, and H12 were all supported. Additionally, control variables do not have a significant impact on medical overuse (Table 4).

**Table 4:** Assessment of the structural model.

Hypothesis	β	STDEV	*T*-statistic	*p*-value	Result
H1: MHIO - > PSEV	0.202	0.030	6.807	0.000	Support
H2: MHIO - > PSUS	0.222	0.028	8.083	0.000	Support
H3: MHIO - > PTB	0.077	0.029	2.631	0.009	Support
H4: MHIO - > PBA	0.191	0.028	6.777	0.000	Support
H5: MHIO - > SE	−0.152	0.029	5.165	0.000	Support
H6: MHIO - > CTA	0.149	0.029	5.130	0.000	Support
H7: PSEV - > MOU	0.246	0.026	9.478	0.000	Support
H8: PSUS - > MOU	0.228	0.025	9.133	0.000	Support
H9: PTB - > MOU	0.046	0.029	1.585	0.113	Reject
H10: PBA - > MOU	0.235	0.026	9.061	0.000	Support
H11:SE - > MOU	−0.145	0.026	5.543	0.000	Support
H12: CTA - > MOU	0.197	0.025	7.998	0.000	Support
Age - > MOU	−0.003	0.027	0.107	0.915	–
Gender - > MOU	0.041	0.054	0.761	0.447	–
Income - > MOU	−0.011	0.027	0.414	0.679	–

MHIO, mHealth information overload; PSEV, perceived severity; PSUS, perceived susceptibility; PTB, perceived treatment benefits; PBA, perceived barriers; SE, self-efficacy; CTA, cues to action; MOU, overuse of health services.

Finally, we used the Standardized Root Mean Square Residual (SRMR) to test the goodness of fit (GOF) of the model. The SRMR value for the model is 0.054, which is below the threshold of 0.08. Thus, the fit of the model is satisfactory (94).

## 5 Discussion

Using the Health belief Model (HBM) as a theoretical framework, this study explores the relationship between information overload in mobile health applications and overuse of medical services, and reveals how information overload affects users’ health behaviors. The findings suggest that information overload significantly enhances users’ perceived severity and susceptibility to health threats, which in turn leads to overuse of medical services. This is consistent with previous research suggesting that in an information-flooded environment, users are more likely to perceive health risks and may therefore take more medical interventions (95).

Secondly, this study found that information overload also had a positive and significant impact on users’ perception of treatment benefits and obstacles. Information overload may enhance users’ beliefs about the benefits of treatment through the placebo effect (47), but this perception does not directly lead to overuse of medical services. We hypothesized that this is not significant because, in the HBM framework, perceived benefit refers to a patient’s attitude toward adopting a particular health behavior when they are aware of a health threat, which is influenced by personal beliefs (96). Overuse of medical services is a condition in which negative effects outweigh positive benefits in health behaviors (2). Based on the contrast between the two, individuals who perceive the benefits of treatment and hold the best beliefs may not be inclined to engage in excessive medical behavior, but rather evaluate their health options through a more deliberate process. In addition, information overload exacerbates users’ concerns about the cost, time, and money of treatment, thereby increasing psychological and practical barriers to treatment and contributing to users’ tendency to over-perform diagnostic tests and treatments (97). This has had a direct impact on the overuse of medical services.

Finally, this study also reveals that there is a positive correlation between information overload and self-efficacy and action cues. More medical information can improve users’ health literacy and enhance their sense of self-efficacy. However, high self-efficacy may drive users to rely more frequently on mobile health applications, increasing the frequency of seeking medical interventions. This finding is also supported by the theoretical impact of HBM’s expanded self-efficacy on health behavior (98). At the same time, the increase of action cues in the context of information overload may lead to excessive medical treatment by users, which indicates that special attention should be paid to how to appropriately provide information prompts when designing mobile medical applications. To avoid triggering unnecessary medical behaviors (99).

This study provides a multi-faceted contribution to the relationship between information overload and overuse of medical services in mHealth apps. (1) In terms of theoretical contributions, this study innovatively applies the health belief model (HBM) to the study of information overload, reveals how information overload affects users’ medical service usage behavior through key elements of HBM, and expands the application of HBM in the field of mobile health care. (2) At the practical application level, this study provides specific recommendations for stakeholders such as mobile health application developers, medical service providers, and policy makers. For example, developers should focus on how to design more intuitive and user-friendly interfaces to ensure that information is accurate and relevant to avoid information overload. Healthcare providers should provide clear guidance during online consultations to help patients sift through important information and avoid misunderstandings or anxiety caused by information overload. Policy makers should develop regulations to ensure the quality of information and prevent medical waste. Users should be rational about the information they get from the app, and learning how to screen and evaluate health information will help avoid excessive medical behavior.

However, there are some limitations to this study. First, the sample is limited to Guangdong Province and mainly focuses on users aged 30 to 49, which may affect the universality of the results. Future studies should expand the geographic and age range of the sample to enhance the external validity of the results. Second, this study mainly used the health belief model (HBM) for analysis, which can be combined with other theoretical models such as planned behavior theory or technology acceptance model in the future to explore more potential factors influencing user behavior. Third, the quantitative methods of information overload and the definition of overuse of medical services can be further refined and discussed in order to enrich the theoretical basis and practical application of related research fields. The fourth is the lack of longitudinal studies. Using a cross-sectional design and collecting data only at specific points in time, this study cannot adequately capture the dynamic between information overload and overuse of healthcare services. Future studies could consider using longitudinal designs to track the evolution of user behavior over time to gain a more complete understanding of the long-term impact of information overload on medical overuse. Finally, there are limitations to the measurement of variables. Although this study used validated scales to measure variables such as information overload, these tools may not fully capture users’ complex psychological responses to information overload. Future research could develop more contextualized and nuanced measurement tools to more accurately assess the impact of information overload on user decision-making and behavior.

## Data Availability

The raw data supporting the conclusions of this article will be made available by the authors, without undue reservation.

## Footnotes

^1^http://index.baidu.com

^2^https://webpower.psychstat.org/
