# Supplementary material for: The paradox of convenience: how information overload in mHealth apps leads to medical service overuse
Source: Front Public Health. 2024 Nov 28;12:1408998. doi: 10.3389/fpubh.2024.1408998 (PMC11634807; doi:10.3389/fpubh.2024.1408998)
Supplement: Supplementary file 1 [file Table_1.docx]

## Appendix A: Measurement scales for variables

| Factors | Serial Number | Item | Reference |
| --- | --- | --- | --- |
| MHealth app Information Overload (MHIO) | MHIO1 | The medical information in mobile healthcare applications is overwhelming. | (Cao et al., 2021) |
|  | MHIO2 | I often spend a significant amount of time searching for information related to my symptoms in these apps. |  |
|  | MHIO3 | The abundance of information in mobile healthcare apps makes it difficult for me to confirm my symptoms. |  |
|  | MHIO4 | The information provided by mobile healthcare apps may lead me to make inappropriate judgments. |  |
| Perceived severity (PSEV) | PSEV1 | After searching for information on mobile healthcare apps, I find myself more concerned about my health. | (Walrave et al., 2020) |
|  | PSEV2 | The thought of possibly having a certain illness makes me anxious. |  |
|  | PSEV3 | By searching for corresponding symptoms, I feel that my condition may be quite serious. |  |
|  | PSEV4 | Based on the information from mobile healthcare apps, I feel that if left untreated, my condition could worsen. |  |
| Perceived susceptibility （PSUS） | PSUS1 | Browsing through mobile healthcare app information, I feel that I am at risk of having a certain illness. | (Walrave et al., 2020) |
|  | PSUS2 | By searching on mobile healthcare apps, I believe I may already have contracted a certain disease. |  |
|  | PSUS3 | Through querying mobile healthcare apps, I believe there is a possibility of developing a certain disease in the future. |  |
|  | PSUS4 | Based on the mobile healthcare information, I perceive a higher likelihood of having a certain disease compared to others. |  |
| Perceived Treatment Benefits (PTB) | PTB1 | I can fulfill my healthcare needs based on the information provided by medical apps. | (Liu et al., 2023) |
|  | PTB2 | Through the information on mobile healthcare apps, I believe I can easily manage my illness. |  |
|  | PTB3 | By adopting healthy behaviors based on the information from mobile healthcare apps, I will not worry too much about my health. |  |
|  | PTB4 | Regularly browsing through the information on mobile healthcare apps makes me feel reassured. |  |
| Perceived Barriers  （PBA） | PBA1 | According to the information from mobile healthcare apps, I believe my illness is difficult to cure. | (Adiyoso et al., 2023) |
|  | PBA2 | Browsing through mobile healthcare information makes me worry about my physical health. |  |
|  | PBA3 | Based on the information from mobile healthcare apps, I perceive my health issues to be rather complex. |  |
|  | PBA4 | According to the information from mobile healthcare apps, I anticipate that curing my illness will take a considerable amount of time. |  |
| Self-efficacy  （SE） | SE1 | I believe that following the information on mobile healthcare apps can resolve my medical issues. | (Wu et al., 2020) |
|  | SE2 | I trust that the medical information in mobile healthcare apps can assist me. |  |
|  | SE3 | I am confident that I will continue to use mobile healthcare apps in the future. |  |
|  | SE4 | I am certain that I will persist in using mobile healthcare apps. |  |
| Cues to Action（CTA） | CTA1 | The information I browse through on mobile healthcare apps prompts me to engage in corresponding health behaviors. | (Arabyat et al., 2023) |
|  | CTA2 | The information I search for on mobile healthcare apps prompts me to undertake relevant medical actions. |  |
|  | CTA3 | If my friends recommend medical information from mobile healthcare apps to me, I will adopt related health behaviors. |  |
|  | CTA4 | If my family recommends medical information from mobile healthcare apps to me, I will adopt relevant medical actions. |  |
| Overuse of health services（MOU） | MOU1 | I prefer treatment options that offer significant efficacy, even if they are less gentle than conservative treatments. | (Ding et al., 2019) |
|  | MOU2 | The more time I spend on mobile healthcare apps, the more medical actions I am inclined to take. |  |
|  | MOU3 | When I feel that my illness might be serious, I always immediately try various health treatment behaviors. |  |
|  | MOU4 | I cannot resist the urge to check medical information on mobile healthcare apps. |  |

**Reference：**

Adiyoso, W., Wilopo, W., Nurbaiti, B., & Suprapto, F. A. (2023). The use of Health Belief Model (HBM) to explain factors underlying people to take the COVID-19 vaccine in Indonesia. *Vaccine: X*, 14, 100297.

Arabyat, R. M., Nusair, M. B., Al-Azzam, S. I., Amawi, H. A., & El-Hajji, F. D. (2023). Willingness to pay for COVID-19 vaccines: Applying the health belief model. Research in Social and Administrative Pharmacy, 19(1), 95-101.

Cao, J., Liu, F., Shang, M., & Zhou, X. (2021). Toward street vending in post COVID-19 China: Social networking services information overload and switching intention. *Technology in Society*, 66, 101669.

Ding, J., Liu, W., Wang, X., Lan, Y., Hu, D., Xu, Y., Li, J., & Fu, H. (2019). Development of a smartphone overuse classification scale. *Addiction Research & Theory*, 27(2), 150-155.

Liu, D., Son, S., & Cao, J. (2023). The determinants of public acceptance of telemedicine apps: an innovation diffusion perspective. *Frontiers in Public Health*, 11:1325031.

Walrave, M., Waeterloos, C., & Ponnet, K. (2020). Adoption of a contact tracing app for containing COVID-19: A health belief model approach. *JMIR Public Health and Surveillance*, 6(3), e20572.

Wu, S., Feng, X., & Sun, X. (2020). Development and evaluation of the health belief model scale for exercise. International Journal of Nursing Sciences, 7, S23-S30.
